# Systemic inflammation response index and Parkinson’s disease risk: A cross-sectional study

**DOI:** 10.1097/MD.0000000000047182

**Published:** 2026-01-23

**Authors:** Weidong Wu, Ying Liu, Shun Gong, Shimiao Wang, Wei Lei, Yingqun Tao

**Affiliations:** a Department of Neurosurgery, General Hospital of Northern Theater Command, Shenyang, Liaoning, China; b Department of Graduate School, China Medical University, Shenyang, Liaoning, China; c Department of Neurology, Shenyang Red Cross Hospital, Shenyang, Liaoning, China.

**Keywords:** cross-sectional study, NHANES, Parkinson’s disease, systemic inflammation response index (SIRI)

## Abstract

Parkinson’s disease (PD) is a highly prevalent neurodegenerative disorder, and inflammation plays a critical role in its pathogenesis. The systemic inflammation response index (SIRI) reflects the overall inflammatory status and has been associated with the development of various diseases. This study aims to investigate the association between the SIRI and PD. Data was obtained from the National Health and Nutrition Examination Survey from 2003 to 2020. A total of 29,022 participants aged ≥40 years were included, of whom 275 were diagnosed with PD. Weighted multivariable logistic regression models were used to evaluate the association between the SIRI and PD, with progressive adjustments for demographic and clinical covariates. Restricted cubic spline regression was used to assess potential nonlinearity, whereas subgroup analyses were used to examine effect modifications. The predictive performance of the SIRI for PD was evaluated via receiver operating characteristic curves. PD patients were older and had higher prevalence of hypertension, diabetes, body mass index, and SIRI level compared with non-PD participants (all *P* < .05). In unadjusted analysis, each unit increase in SIRI was associated with higher odds of PD (odds ratio = 1.208, 95% confidence interval: 1.112–1.313, *P* < .001). The association remained significant after full adjustment (odds ratio = 1.162, 95% confidence interval: 1.051–1.285, *P* = .004). Restricted cubic spline analysis revealed a positive association between the SIRI and PD prevalence (*P*-overall < .001), with evidence of a nonlinear trend (*P*-nonlinear = .047). Subgroup analyses showed consistent results across age, sex, race, education level, body mass index, hypertension, diabetes, and smoke, with no significant interactions. The predictive ability of the SIRI for PD was modest, with an area under the receiver operating characteristic curve (area under the curve) of 0.579. Our findings demonstrate a significant dose–response association between the SIRI and PD. This highlights the potential utility of the SIRI as a predictive biomarker, which may be incorporated into screening frameworks to facilitate early detection and preventive interventions.

## 1. Introduction

Parkinson’s disease (PD) is the second most common neurodegenerative disorder worldwide, characterized by progressive motor dysfunction, as resting tremors, rigidity and bradykinesia.^[1]^ The pathology is characterized by gradual degeneration of dopamine (DA) neurons in the substantia nigra.^[2]^ With global population aging, the prevalence of PD is expected to increase, creating considerable challenges for patients, caregivers, and healthcare systems worldwide.^[3]^ Therefore, identifying potential risk factors for Parkinson’s disease is crucial for early detection and prevention strategies.

Growing evidence suggests that chronic systemic inflammation plays a crucial role in PD pathogenesis.^[4,5]^ The systemic inflammation response index (SIRI), which is calculated from neutrophil, monocyte, and lymphocyte counts, has emerged as a novel biomarker reflecting the systemic inflammatory status. Elevated SIRI has been associated with cardiovascular disease, malignancies, and metabolic disorders, but its relationship with PD remains unclear.^[6–8]^

This study aimed to investigate the relationship between the SIRI and PD using data from the National Health and Nutrition Examination Survey (NHANES) from 2003 to 2020. By investigating these associations, this study aims to provide evidence for the role of systemic inflammatory responses in the development of PD and to offer potential implications for early identification and prevention strategies in high-risk populations.

## 2. Materials and methods

### 2.1. Study design and population

**Figure 1.**
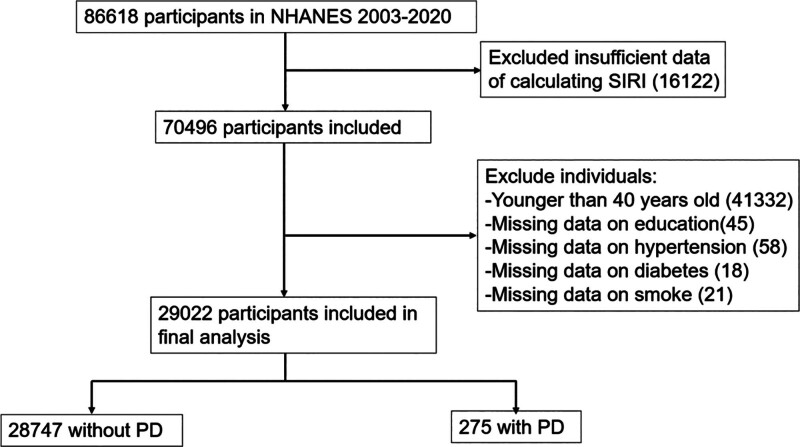
Flowchart of study participants. NHANES = National Health and Nutrition Examination Survey, PD = Parkinson’s disease, SIRI = systemic inflammation response index.

Since 1999, the NHANES, administered by the National Center for Health Statistics of the CDC, has continuously gathered information on demographic characteristics, socioeconomic factors, health status, and dietary habits from a representative sample of the US population. This cross-sectional study, we utilized data from participants in the 2003 to 2004, 2005 to 2006, 2007 to 2008, 2009 to 2010, 2011 to 2012, 2013 to 2014, 2015 to 2016, and 2017 to 2020 survey cycles, comprising a total of 86,618 individuals. After excluding respondents younger than 40 years and those with incomplete survey information, 29,022 participants were included in the final analysis (Fig. 1).

### 2.2. Definition of PD

Parkinson’s disease status in the NHANES was determined through self-reported prescription medication data, focusing on drugs listed under the “Anti-Parkinson Agents” category. Participants who reported current use of such medications were classified as having PD, whereas those without corresponding prescriptions were considered non-PD. This operational definition aligns with approaches adopted in prior research.^[9–12]^

### 2.3. Definition of the systemic inflammation response index

In accordance with previous studies,^[13,14]^ we calculated the SIRI via the following formula:


$$\begin{array}{l} SIRI=\frac{Neutrophil\;count\times Monocyte\;count}{Lymphocye\;count} \end{array}$$

The neutrophil, monocyte, and lymphocyte counts were obtained from the complete blood count (CBC) data in NHANES. CBC measurements in the NHANES were performed via the Beckman Coulter system, which applies impedance and optical methods with an automated dilution and mixing device for sample preparation. Detailed protocols for these laboratory assessments are available in the NHANES Laboratory/Medical Technician Procedure Manual.

### 2.4. Assessment of covariates

We included the following covariates: age, gender (male, female), race (Mexican American, other Hispanic, non-Hispanic White, non-Hispanic Black, other races including multi-racial), education (less than high school, high school or equivalent, college or above), body mass index (BMI), hypertension (yes, no), diabetes (yes, no), smoke (yes, no). Previous disease diagnosis information was based on self-reported information.

### 2.5. Statistical analysis

Because the NHANES adopts a complex, multistage probability sampling design, we applied 2-year MEC examination weights (WTMEC2YR) for all weighted analyses. The SIRI was first calculated, after which descriptive statistics were generated for the overall cohort and for stratified groups. Continuous variables were summarized as the means with standard deviations, while categorical variables were presented as counts and percentages. The Kolmogorov–Smirnov test was applied to assess the normality of the distributions. Group comparisons were performed using the chi-square test for categorical variables, analysis of variance or independent sample *t* tests for normally distributed continuous variables, and the Mann–Whitney *U* test for non-normally distributed continuous variables. To examine the association between the SIRI and Parkinson’s disease, we constructed 3 logistic regression models. Model 1 was unadjusted. Model 2 included adjustments for demographic factors (age, sex, race/ethnicity, and education level). Model 3 further incorporated BMI, hypertension, diabetes, and smoke as covariates. Subgroup analyses were subsequently performed to assess potential effect modifications, with 1 model fitted per stratification variable while adjusting for the remaining covariates. To formally test interaction effects, interaction terms between the SIRI and each stratification variable were added to the models. Individuals with missing covariate data were excluded, and sensitivity analyses were carried out to assess the robustness of the findings. Additionally, restricted cubic spline (RCS) regression, adjusted for all covariates in model 3, was employed to evaluate the potential nonlinear relationship between the SIRI and PD. The diagnostic performance of the SIRI for predicting PD was further assessed using receiver operating characteristic (ROC) curves and the area under the curve (AUC). All analyses were performed in R (version 4.3.3), with 2-sided *P* < .05 considered statistically significant. The results are presented as odds ratios with 95% confidence intervals (CIs).

## 3. Results

### 3.1. Characteristics of study participants

Table 1 presents the differences in patient characteristics between PD patients and non-PD individuals. A total of 29,022 participants were included in this study, of whom 275 individuals had PD. Compared with non-PD participants, PD patients were older (64.84 vs 59.98 years, *P* < .001). In terms of racial distribution, non-Hispanic Whites accounted for the highest proportion of PD patients (69.8%), while non-Hispanic Blacks (12.0%) and Mexican Americans (8.4%) had lower proportions (*P* < .001). In terms of education level, some college or AA degree accounted for the highest proportion among PD patients (32.0%), while high school graduate (21.5%), college graduate or above (19.6%), and 9 to 11th grade (17.5%) had lower proportions (*P* = .049). Regarding health conditions, PD patients had a higher prevalence of hypertension (60.0%, *P* < .001), a higher prevalence of diabetes (24.4%, *P* = .028), a higher BMI (30.47 ± 6.85, *P* = .019), and a higher SIRI (1.54 ± 1.12, *P* < .001). Additionally, there were no significant differences between PD patients and non-PD individuals in terms of gender, smoke (*P* > .05). In summary, these findings suggest that PD is associated with older age, specific racial groups, education level, hypertension, diabetes, higher BMI, and elevated SIRI.

**Table 1:** Characteristics of participants grouped by Parkinson’s disease in NHANES 2003–2020.

Variables	Total	Non-PD	PD	*P*-value
N	29,022	28,747	275	
Age (mean [SD])	60.03 (12.46)	59.98 (12.45)	64.84 (12.70)	**<.001**
Gender, n (%)				.069
Female	14,829 (51.1)	14,673 (51.0)	156 (56.7)	
Male	14,193 (48.9)	14,074 (49.0)	119 (43.3)	
Race, n (%)				**<.001**
Mexican American	4160 (14.3)	4137 (14.4)	23 (8.4)	
Non-Hispanic Black	6251 (21.5)	6218 (21.6)	33 (12.0)	
Non-Hispanic White	13,153 (45.3)	12,961 (45.1)	192 (69.8)	
Other Hispanic	2619 (9.0)	2605 (9.1)	14 (5.1)	
Other race – including multi-racial	2839 (9.8)	2826 (9.8)	13 (4.7)	
Education, n (%)				**.049**
9–11th grade	4001 (13.8)	3953 (13.8)	48 (17.5)	
College graduate or above	6430 (22.2)	6376 (22.2)	54 (19.6)	
High school graduate	6775 (23.3)	6716 (23.4)	59 (21.5)	
<9th grade	3905 (13.5)	3879 (13.5)	26 (9.5)	
Some college or AA degree	7911 (27.3)	7823 (27.2)	88 (32.0)	
BMI (mean [SD])	29.52 (6.75)	29.51 (6.75)	30.47 (6.85)	**.019**
Hypertension, n (%)				**<.001**
No	15,052 (51.9)	14,942 (52.0)	110 (40.0)	
Yes	13,970 (48.1)	13,805 (48.0)	165 (60.0)	
Diabetes, n (%)				**.028**
Borderline	855 (2.9)	847 (2.9)	8 (2.9)	
No	22,885 (78.9)	22,685 (78.9)	200 (72.7)	
Yes	5282 (18.2)	5215 (18.1)	67 (24.4)	
Smoke, n (%)				.708
No	14,942 (51.5)	14,804 (51.5)	138 (50.2)	
Yes	14,080 (48.5)	13,943 (48.5)	137 (49.8)	
SIRI (mean [SD])	1.29 (0.98)	1.28 (0.98)	1.54 (1.12)	**<.001**

BMI = body mass index, PD = Parkinson’s disease, SD = standard deviation, SIRI = systemic inflammation response index.

### 3.2. Association between the SIRI and PD

Table 2 summarizes the association between the SIRI and PD across different models. In model 1, which was unadjusted for covariates, each 1-unit increase in the SIRI as a continuous variable was associated with a 20.8% increase in PD risk (odds ratio [OR] = 1.208, 95% CI: 1.112–1.313, *P* < .001). In model 2, which was adjusted for age, sex, race, education level the OR for SIRI as a continuous variable decreased to 1.169 (95% CI: 1.063–1.286, *P* = .002). In model 3, which was fully adjusted for all covariates, including BMI, hypertension, diabetes, and smoke, the OR for the use of the SIRI as a continuous variable was 1.162 (95% CI: 1.051–1.285, *P* = .004). These findings suggest that a higher SIRI is correlated with increased odds of PD, decreasing from an OR of 1.208 (unadjusted) to an OR of 1.162 (fully adjusted) while retaining statistical significance.

**Table 2:** The Associations of the SIRI and Parkinson’s disease in weighted logistic regression models.

Variables	Model 1		Model 2		Model 3	
	OR (95% CI)	*P*-value	OR (95% CI)	*P*-value	OR (95% CI)	*P*-value
SIRI	1.208 (1.112–1.313)	**<.001**	1.169 (1.063–1.286)	**.002**	1.162 (1.051–1.285)	**.004**

CI = confidence interval, OR = odds ratio, SIRI = systemic inflammation response index.

### 3.3. The associations between the SIRI and PD in RCS regression model

**Figure 2.**
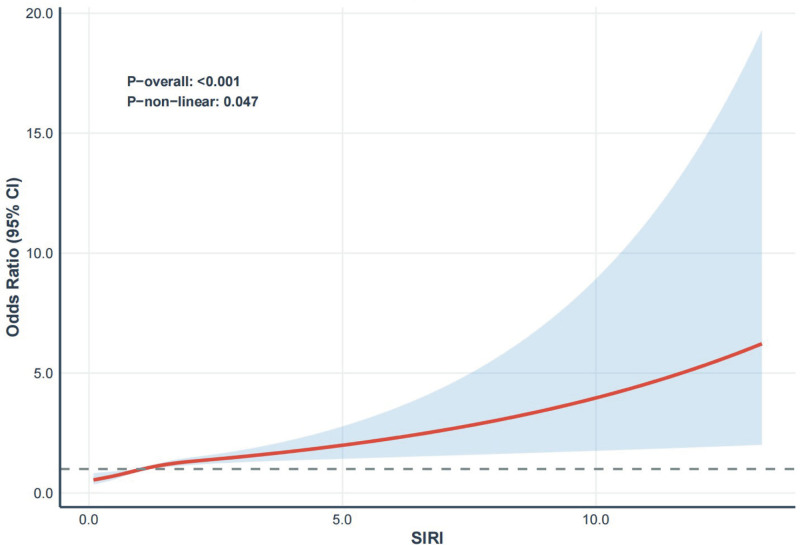
Restricted cubic spline (RCS) curve of the association between systemic inflammation response index (SIRI) and Parkinson’s disease (PD) among all participants. The red solid line indicates the estimated odds ratios (ORs) for PD across the spectrum of SIRI, with the blue shaded area representing the 95% confidence intervals (CIs). The gray dashed horizontal line represents the null value (OR = 1.0). The association between the SIRI and PD was statistically significant overall (*P*-overall < .001), with a marginally significant nonlinear trend (*P*-nonlinear = .047). CI = confidence interval, OR = odds ratio, PD = Parkinson’s disease, RCS = restricted cubic spline, SIRI = systemic inflammation response index.

The dose–response analysis revealed a significant positive association between SIRI and PD (*P*-overall < .001), with suggestive evidence of a nonlinear trend (*P*-nonlinear = .047; Fig. 2). With increasing SIRI levels, the likelihood of PD increased accordingly, suggesting that the SIRI may serve as an important indicator of PD.

### 3.4. Subgroup analysis

**Figure 3.**
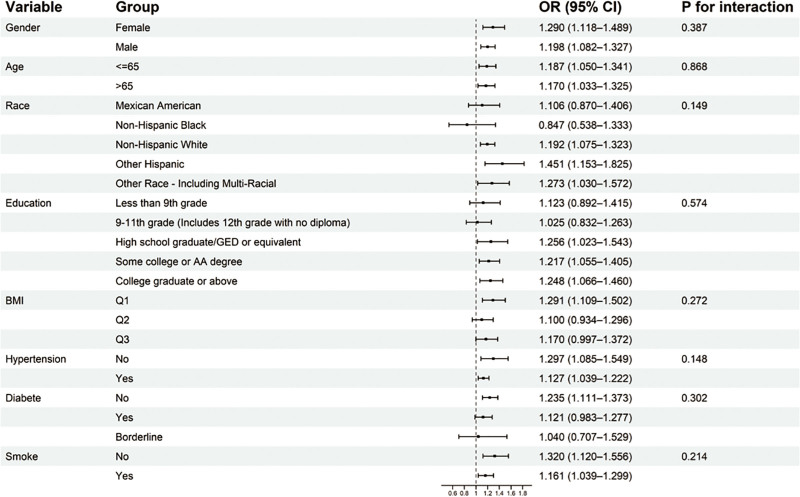
Subgroup analyses of the associations between the systemic inflammation response index (SIRI) and Parkinson’s disease (PD). This forest plot displays the odds ratios (ORs) and 95% confidence intervals (CIs) for the association between the SIRI and PD across different subgroups. Subgroups include age, sex, race, education level, body mass index (BMI) level, hypertension, diabetes and smoke. *P* values for interaction were calculated to assess effect modification. BMI = body mass index, CI = confidence interval, OR = odds ratio, PD = Parkinson’s disease, SIRI = systemic inflammation response index.

Subgroup analyses were conducted according to age, sex, race, education level, BMI level, hypertension, diabetes and smoke (Fig. 3). The positive association between the SIRI and PD remained generally consistent across these subgroups. Notably, none of the interaction tests reached statistical significance (all *P* for interaction > .05), suggesting that the observed association was stable and did not differ substantially across different demographic and clinical strata.

### 3.5. Predictive value of the SIRI for PD

**Figure 4.**
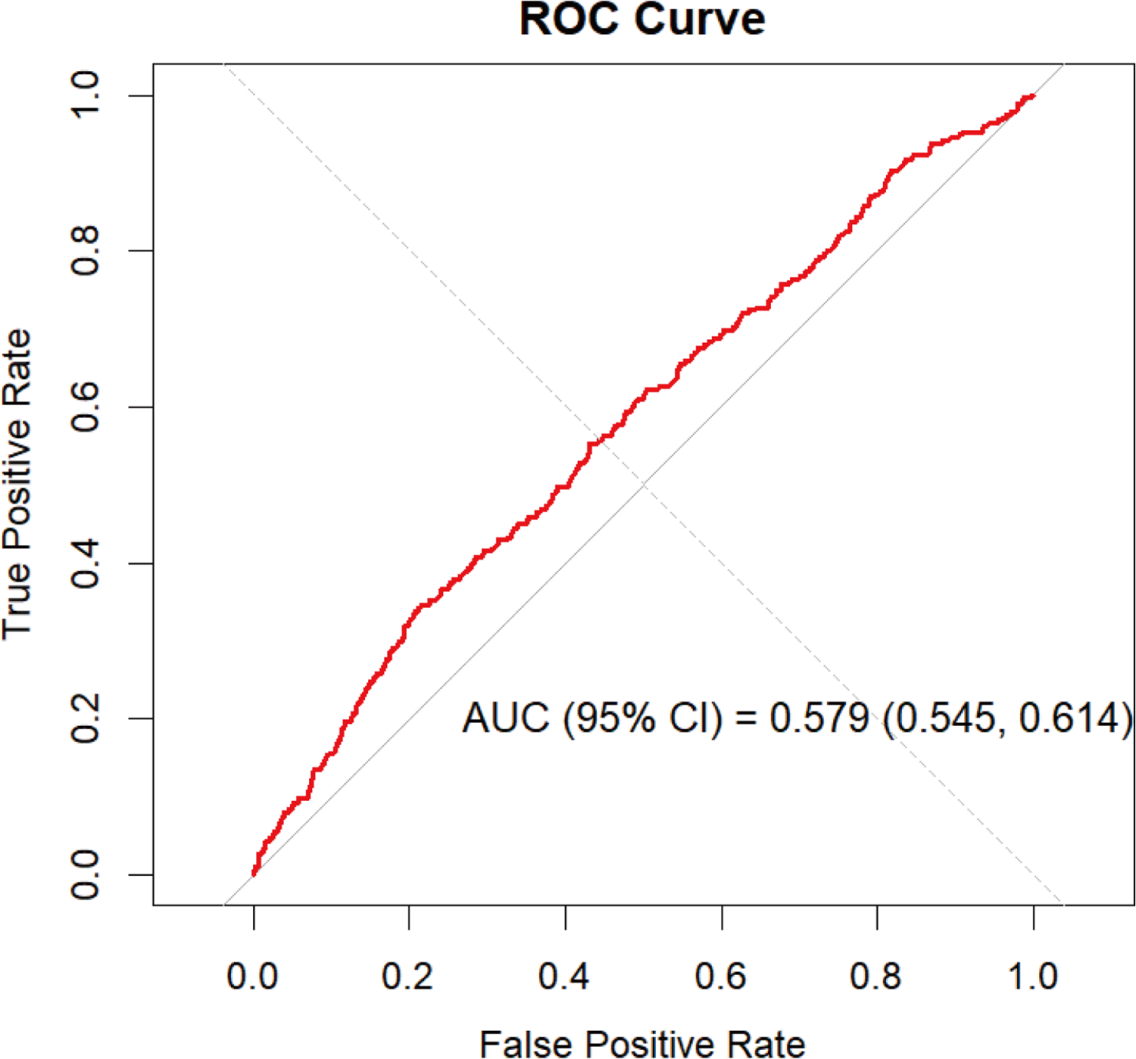
Receiver operating characteristic (ROC) curve evaluating the predictive performance of the systemic inflammation response index (SIRI) for Parkinson’s disease (PD). The ROC curve (red line) illustrates the trade-off between the sensitivity and specificity of the SIRI in predicting PD. The area under the curve (AUC) was 0.579 (95% CI: 0.545–0.614), indicating poor discriminatory ability. AUC = area under the curve, PD = Parkinson’s disease, ROC = receiver operating characteristic, SIRI = systemic inflammation response index.

We evaluated the predictive performance of the SIRI for PD risk using ROC curve analysis. The results showed that the AUC was 0.579 (Fig. 4), indicating limited standalone discriminative capacity. Although the AUC suggests suboptimal predictive power, the observed association between the SIRI and PD highlights its potential role as a complementary marker within multiparameter risk assessment models.

## 4. Discussion

This study, which utilized NHANES data from 2003 to 2020, provides nationally representative evidence from the US adult population. We found that elevated SIRI levels were positively associated with PD prevalence, and the association persisted after adjusting for demographic and clinical covariates. Although the SIRI may serve as a potential marker for identifying individuals at greater risk of PD, the cross-sectional design precludes causal inference. Hence, these findings should be interpreted with caution, and prospective studies are needed to confirm the observed associations.

Our findings differ partly from those of Wang et al who found no significant relationship between SIRI and PD after full adjustment.^[15]^ Our analysis incorporated more recent NHANES cycles (2003–2020), thereby enhancing population representativeness. We further treated SIRI as a continuous variable and examined its association with PD using RCS models, which revealed a positive linear trend. In addition, subgroup and ROC analyses were performed to evaluate effect modification and predictive ability. Overall, our study refines and extends previous findings by providing updated and more comprehensive evidence that elevated SIRI is independently associated with PD. Numerous studies have demonstrated that peripheral inflammatory markers are closely involved in the pathogenesis and progression of PD.^[16,17]^ A study by Muñoz-Delgado revealed that a lower lymphocyte count was significantly associated with lower dopamine transporter levels in both the caudate and the putamen, suggesting a relationship between systemic inflammation and dopaminergic degeneration in patients with PD.^[18]^ A meta-analysis showed that an increased levels of neutrophil-to-lymphocyte ratio is strongly related to the presence of PD.^[19]^ Another meta-analysis by Qu demonstrated that patients with PD exhibited elevated levels of interleukin-6 (IL-6), Tumor necrosis factor alpha (TNF-α), interleukin-1beta, soluble tumor necrosis factor receptor-1, C-reactive protein, C-C motif ligand 2, C-X3-C motif chemokine ligand 1, and C-X-C motif ligand 12 in peripheral blood, as well as increased cerebrospinal fluid concentrations of IL-6, TNF-α, interleukin-1beta, C-reactive protein, and C-C motif ligand 2 compared with controls, suggesting that PD is associated with inflammatory responses in both the peripheral circulation and the cerebrospinal fluid.^[20]^ The SIRI, which integrates neutrophils, monocytes, and lymphocytes into a single index, may provide a more comprehensive reflection of systemic inflammatory status than any single ratio. Our results extend prior observations by highlighting the SIRI as a potential inflammatory biomarker linked to PD. A higher SIRI was positively associated with PD, and although the effect size attenuated after adjustment for covariates, the association remained statistically significant.

Several biological mechanisms may explain the observed association. Chronic neuroinflammation is a well-recognized feature of PD and is characterized by microglial activation, oxidative stress, and proinflammatory cytokine release, which collectively contribute to dopaminergic neuronal damage.^[21–23]^ Peripheral immune dysregulation may further exacerbate central inflammation through disruption of the blood–brain barrier, recruitment of activated monocytes, and cross-talk between systemic and central immune responses.^[5,24,25]^ Recent studies suggest that systemic inflammation contributes to PD pathogenesis through immune-neural interactions. Systemic cytokines such as TNF-α and IL-6 may disrupt the blood–brain barrier, allowing immune cells to infiltrate the CNS and activate microglia. Activated microglia and astrocytes release neurotoxic mediators that promote dopaminergic neuronal loss. Reduced regulatory T-cell activity further sustains a proinflammatory state, while α-synuclein aggregates can amplify immune activation, forming a vicious feedback loop between peripheral and central inflammation. This immune–inflammatory cascade likely underlies both neurodegeneration and cognitive impairment in PD and highlights inflammation as a potential target for disease modification.^[26]^ Since the SIRI captures both proinflammatory (neutrophils, monocytes) and anti-inflammatory (lymphocytes) components, higher SIRI values may indicate a shift toward a proinflammatory state, thereby reflecting the underlying biological processes that predispose individuals to PD.

The SIRI represents a simple, inexpensive, and readily available biomarker that can be derived from routine CBCs. The SIRI integrates neutrophils, monocytes, and lymphocytes, providing a more comprehensive reflection of the systemic inflammatory status.^[27]^ In peritoneal dialysis patients, the SIRI has been shown to predict all-cause and cardiovascular mortality comparably or better than the neutrophil-to-lymphocyte ratio and the monocyte-to-lymphocyte ratio.^[28]^ In the subgroup analyses none of the interaction tests reached statistical significance (all *P* for interaction > .05), suggesting that the observed association was stable and did not differ substantially across different demographic and clinical strata. Although our ROC analysis indicated the limited discriminatory ability of the SIRI alone (AUC = 0.579), its significant association with PD suggests that it may serve as a complementary marker within multiparameter risk assessment models. Given its simplicity and availability from routine blood tests, the SIRI could be incorporated into screening or risk stratification frameworks to facilitate early monitoring and preventive interventions among high-risk populations.

Nevertheless, several limitations should be acknowledged. First, the cross-sectional design of the NHANES precludes causal inference, and reverse causation cannot be excluded. Second, identifying PD through antiparkinsonian drug use may fail to distinguish it from other parkinsonian disorders, possibly leading to misclassification and underestimation of PD prevalence, particularly in untreated cases. Third, although we adjusted for multiple covariates, residual confounding from unmeasured factors cannot be ruled out. Moreover, the low AUC value indicates that the SIRI alone has limited predictive performance for Parkinson’s disease and should not be used in isolation for clinical decisions. Finally, the number of PD patients in our study was relatively small compared with that in the non-PD group, which may have limited the statistical power of the subgroup analyses.

## 5. Conclusion

In conclusion, our study demonstrated that the higher SIRI levels are significantly associated with PD in the United State adult population. Moreover, the predictive performance of the SIRI was modest (AUC = 0.579), suggesting that the SIRI should be considered a supplementary, rather than standalone, risk indicator for PD. These findings highlight the potential role of systemic inflammation in PD and suggest that the SIRI may serve as a useful biomarker in the context of multifactorial risk assessment, although further longitudinal and mechanistic research is warranted.

## Acknowledgments

We thank NCHS for providing the NHANES data. We also acknowledge the participants and staff involved in NHANES for their valuable contributions to this research.

## Author contributions

**Conceptualization:** Yingqun Tao.

**Data curation:** Shun Gong, Yingqun Tao.

**Formal analysis:** Shun Gong, Yingqun Tao.

**Funding acquisition:** Shun Gong, Yingqun Tao.

**Investigation:** Shun Gong, Yingqun Tao.

**Methodology:** Shun Gong, Yingqun Tao.

**Project administration:** Shimiao Wang, Wei Lei.

**Resources:** Shun Gong, Yingqun Tao.

**Software:** Shun Gong, Yingqun Tao.

**Supervision:** Yingqun Tao.

**Validation:** Shun Gong, Yingqun Tao.

**Visualization:** Shun Gong, Yingqun Tao.

**Writing – original draft:** Weidong Wu, Ying Liu.

**Writing – review & editing:** Weidong Wu, Ying Liu.
